# Family Commitment and Work Characteristics among Pharmacists

**DOI:** 10.3390/pharmacy3040386

**Published:** 2015-12-17

**Authors:** Paul O. Gubbins, Denise Ragland, Ashley N. Castleberry, Nalin Payakachat

**Affiliations:** 1 Division of Pharmacy Practice & Administration, University of Missouri-Kansas City, UMKC School of Pharmacy at MSU, 327 West Mill Street, #425 Springfield, MO 65806, USA; E-Mail: gubbinsp@umkc.edu; 2 Department of Pharmacy Practice, University of Arkansas for Medical Sciences, College of Pharmacy, 4301 West Markham, #522, Little Rock, AR 72205, USA; E-Mail: dragland@uams.edu; 3 Department of Pharmaceutical Sciences, University of Arkansas for Medical Sciences, College of Pharmacy, 4301 West Markham, #522, Little Rock, AR 72205, USA; E-Mail: ancastleberry@uams.edu; 4 Division of Pharmaceutical Evaluation and Policy, Department of Pharmacy Practice, University of Arkansas for Medical Sciences, College of Pharmacy, 4301 West Markham, #522, Little Rock, AR 72205, USA

**Keywords:** Professional practice, workforce, work-life benefits, work-life balance, retention

## Abstract

Factors associated with family commitment among pharmacists in the south central U.S. are explored. In 2010, a cross-sectional mailed self-administered 70 item survey of 363 active licensed pharmacists was conducted. This analysis includes only 269 (74%) participants who reported being married. Outcome measures were family commitment (need for family commitment, spouse’s family commitment), work-related characteristics (work challenge, stress, workload, flexibility of work schedule), and job and career satisfaction. Married participants’ mean age was 48 (SD = 18) years; the male to female ratio was 1:1; 73% worked in retail settings and 199 (74%) completed the family commitment questions. Females reported a higher need for family commitment than males (*p* = 0.02) but there was no significant difference in satisfaction with the commitment. Work challenge and work load were significantly associated with higher need for family commitment (*p* < 0.01), when controlled for age, gender, number of dependents, work status, and practice setting. Higher work challenge was associated with higher career satisfaction. Higher job related stress was associated with lower job satisfaction. High work challenge and work load may negatively impact family function since married pharmacists would need higher family commitment from their counterparts. The impact of work-family interactions on pharmacy career satisfaction should be further investigated.

## 1. Introduction

Work and family are important aspects of life, and provide meaning and identity to which individuals balance commitment, or the ties that link them to social structure through the roles, organizations, individuals, and values with which they affiliate [1,2]. Family commitment is a strong personal dedication to a decision to be active/involved in family activities and participate in moral and educational development of children [1]. This commitment is initiated and sustained by the extent to which the individual’s identification with it is central (*i.e.*, significant or meaningful) among their personal hierarchy of identities or self-meanings [1].

Balancing the dual roles of work and family has been a part of everyday life for millions of adults for several decades [1,2]. Thus, research examining the relationship between work and family has primarily focused on the conflict between these domains [2]. The “work-family conflict” is defined as “a form of inter-role conflict in which the role pressures from the work and family domains are mutually incompatible in some respect”, and it can lead to poor individual health and organizational outcomes [2,3]. The role pressures involved are directional and produce negative effects from one domain to the other, which results in two distinct but related constructs [2,4]. Much of the research in the field has examined one construct termed the “work-to-family conflict” (WFC), which occurs when participation in the work role makes participating in the family role difficult [4]. The other construct (*i.e.*, “family-to-work conflict” (FWC)) occurs when participation in the family role makes participating in the work role more difficult [2]. Studies examining the work-family conflict indicate that work role stressors, work role involvement, work social support, work characteristics, and personality are all precursors of the WFC; whereas family role stressors, family social support, family characteristics, and personality are all precursors of the FWC [2]. Moreover, family role stressors (e.g., family stressors, role conflict, role ambiguity, role overload), family involvement (e.g., family interest/centrality), family social support (e.g., family support, spousal support), and family characteristics (family climate) are predictors of WFC; whereas work role stressors (e.g., job stressors, role conflict, role ambiguity, role overload) and work social support (e.g., organizational support, supervisor support, coworker support) are predictors of FWC [2].

In addition to influencing work-related outcomes, the work-family conflict can negatively impact non-work related outcomes including life, marital, family and leisure satisfaction as well as family performance (*i.e.*, domestic labor) [4]. A comprehensive review of outcomes of associated with the WFC evaluated many studies that have examined the conflict and its association with non-work related outcomes, but none were specific to the pharmacy profession [4]. Domestic labor is comprised of housework, child care, and emotional work (*i.e*., providing emotional support) [5]. Providing emotional support is an important aspect of domestic labor. However, traditional domestic labor theories based on gender ideologies, time availability, relative resources and demand-response are not predictive of emotion-work performance [5].

U.S. pharmacists believe that work/life balance is important for career success [6]. However, like any paid employment in the U.S. or in other countries, work place pressures can also produce job stress that negatively affect pharmacy professionals’ health, their workplace, and indirectly, their patients [7,8,9,10,11,12,13,14,15]. National surveys of U.S. pharmacists’ work attitudes also continue to indicate high levels of work-family conflict in nearly all practice settings suggesting work place pressures also negatively affect their family domain [9,16]. The relationship between job stress and work-related attitudes or its impact on the work/life balance have been widely explored in many health care fields, but data specific to the pharmacy profession on many of these issues, such as family commitment are sparse [7,17,18,19,20,21,22].

## 2. Experimental Section

### 2.1. Objective

The objective of this study was to explore associations of family commitment, work-related issues (*i.e.*, stress and workload), and personal characteristics among licensed pharmacists located in the south-central region of the U.S.

### 2.2. Study Design and Participants

A cross-sectional, self-administered mailed survey was conducted for this study. The sampling frame was 41,599 pharmacists with active licenses for the year 2010 in the U.S. South-Central region that includes Alabama, Arkansas, Kentucky, Louisiana, Mississippi, Oklahoma, Tennessee, and Texas. The list of pharmacists was obtained through the State Boards of Pharmacy for all states except for Alabama, which was supplied by LM Lists (Krejcie and Morgan, West Marlton, NJ, USA). Further details of the study are described in a previous publication [23].

The sample size of 382 was estimated to represent this population [24]. A stratified random sampling method was utilized based on the relative numbers of active licensed pharmacists located in the eight states. A total of 2000 surveys were mailed using a modified Dillman’s method [25]. The survey package included a personalized cover letter explaining the purpose of the research project, a numbered survey instrument, and a postage paid return envelope. The packets were mailed in March 2010, and a reminder postcard was sent 1 week later. One month after the first mailing, a second survey packet was sent to non-respondents, followed by a reminder postcard 1 week later. Numbered survey instruments enabled investigators to keep individual data confidential while tracking response rates. Survey instruments that were returned within 2 months of the first mailing date were included in the analysis. Identifiable information, such as name, address, license number, or phone number, was not collected. Surveys that were returned within 2 months of the first mailing date were included in the analysis. This research protocol was approved by the University’s Institutional Review Board on 4 February 2010 (IRB #12735).

### 2.3. Survey

The 70-item survey was categorized into 4 general components: (1) demographics including family commitment; (2) work-related characteristics [26]; (3) preceptorship [26]; and (4) personal perspectives on pharmacy job/career, including satisfaction [27]. The survey instrument was tested for reliability and validity using a structural equation modeling approach [12,15]. Job and career satisfaction domains showed high reliability with Cronbach’s alphas > 0.85. The survey took approximately 10–15 min to complete. Table 1 presents the survey items for this current study. Only job/career satisfaction components were calculated from multiple items. Results of preceptorship and its associations to satisfaction were reported elsewhere [23].

Only participants who had children were instructed to complete the family commitment section. The current research included only married participants because the investigators believed their responses would allow a more direct exploration of the associations of family commitment (*i.e.*, a decision to be active/involved in family activities and participate in moral and educational development of children), work-related issues and personal characteristics of licensed pharmacists, than if individuals who were not married or had never been married at the time of the survey were also included. The beginning of this section provided several examples of family commitment such as scheduling sufficient time and energy for the moral and educational development of the children, reading to them, taking them on frequent outings, developing skill in appropriate child-training methods and discussing child-rearing methods with spouse. The two main items in this section were: (1) need for family commitment (0 = I have no need for family commitment; 6 = I have a great need for family commitment); and (2) evaluation of spouse’s family commitment (0 = I am extremely dissatisfied; 6 = I am extremely satisfied). The need for family commitment also included two items asking how much time the participants would like their spouse to be engaged in family commitment and how they feel if their spouse did not engage in family commitment (1 = very unhappy, 2 = somewhat unhappy, 3 = neither happy nor unhappy).

Four items categorized as work-related characteristics were used to explore associations with family commitment. These included: (1) work challenge (1 = seldom challenged; 4 = highly challenged); (2) work-related stress (1 = not at all stressed; 4 = highly stressed); (3) excessive workload (1 = strongly disagree; 5 = strongly agree); and (4) flexibility of work schedule (1 = strongly disagree; 5 = strongly agree). Items capturing job and career satisfaction comprised the third and fourth categories in Table 1.

**Table 1:** Survey components.

Components	Items	Response Options
*Family Commitment*		
Need for family commitment	Q5A: Indicate how much you need family commitment.	0 = I have no need for family commitment, 3 = I have a moderate need for family commitment, 6 = I have a great need for family commitment
	How much time would you like your spouse to be engaged in family commitment?	Fill in the blank a number of hours per week.
	If your spouse does not spend as much time engaged in family commitment as you indicated above, how does it make you feel?	1 = very unhappy, 2 = somewhat unhappy, 3 = neither happy nor unhappy
Evaluation of spouse’s family commitment	Q5B: Indicate hour satisfaction with your spouse’s family commitment.	0 = I am extremely dissatisfied, 3 = I am neither satisfied nor dissatisfied, 6 = I am extremely satisfied.
*Work-related Characteristics*		
Work challenge	Q15: To what extent do you feel professionally challenged by your work?	1 = seldom challenged, 2 = occasionally challenged, 3 = challenged, 4 = highly challenged
Stress	Q17: How stressed do you feel during an average workday?	1 = not at all stressed, 2 = not too stressed, 3 = somewhat stressed, 4 = highly stressed
Workload	Q44: My workload is excessive.	1 = strongly disagree, 2 = tend to disagree, 3 = neither agree nor disagree, 4 = tend to agree, 5 = strongly agree
Flexibility of work schedule	Q45: My work schedule is flexible.	1 = strongly disagree, 2 = tend to disagree, 3 = neither agree nor disagree, 4 = tend to agree, 5 = strongly agree
*Job and Career Satisfaction*		
Job satisfaction	Q51: All things considered, I am satisfied with my current job.	1 = strongly disagree, 2 = tend to disagree, 3 = neither agree nor disagree, 4 = tend to agree, 5 = strongly agree
	Q52: The idea of spending the remainder of my working life in a job like my current one is depressing.	
	Q53: I often leave work with a “bad” feeling, a feeling that I am doing something which I do not enjoy.	
Career satisfaction	Q56: Knowing what I know now, if I had to decide all over again whether to go into pharmacy, I would choose another field.	1 = strongly agree, 2 = tend to agree, 3 = neither agree nor disagree, 4 = tend to disagree, 5 = strongly disagree
	Q57: If I had a *son* who told me he was interested in pursuing a career in pharmacy, I would encourage him.	
	Q58: If I had a *daughter* who told me she was interested in pursuing a career in pharmacy, I would encourage her.	
	Q59: If I were free to pursue any type of career I wanted I would stay in pharmacy.	

### 2.4. Analyses

Descriptive statistics of married participants were obtained. Although this analysis focused on family commitment regarding perspectives on raising children, it utilized all the responses from married participants whether or not they had children. We explored relationship between age and need of family commitment using Spearman correlation as we expected that young couples with children might need higher family commitment. The sample’s family commitment average score was used to identify high (higher than the average) and low (equal or lower than the average) need for family commitment. The same principle was applied to work challenge, work-related stress, work load, and flexibility of work schedule. The reason for not using the actual scores of the items above was because their response scale was an ordinal scale. In addition, we wanted to explore factors associated with the high groups. Associations of family commitments with job satisfaction, career satisfaction, and participants’ demographics were explored using Wilcoxon-Mann-Whitney or Kruskal-Wallis rank tests, as appropriate. Logistic regression was employed to examine variables associated with high need for family commitment. The criterion for significance in this study was set at an alpha level of 0.05. All statistical analyses were conducted in Stata/SE 13.0 (StataCorp LP, College Station, TX, USA).

## 3. Results

A total of 369 surveys were received which yielded an 18.5% response rate. Six responses were excluded due to retirement (4), unemployment (1), and not practicing pharmacy (1), leaving 363 responses for analysis. The participants’ mean years of practicing pharmacy since receiving their license was 22.9 (SD = 14.8). Sixty percent (*n* = 217) had a B.S. degree, 40% (*n* = 144) had a Pharm.D. degree, and 47% (*n* = 169) had some type of advanced training (e.g., residency, fellowship, certification). Full descriptive statistics of the whole population were previously reported [23].

Table 2 provides the demographics of the married participants. Among the 363 participants, 269 (74%) were married and had an average age of 48 (SD = 14) years. The male to female ratio was 1:1 in the married group. Two thirds of the married participants reported having B.S. degree and 58% worked in retail settings. Table 3 summarizes married participants’ responses regarding family commitment, job satisfaction and career satisfaction as categorized by their demographics. A total of 198 participants completed the family commitment questions. Females reported a higher need for family commitment than males (5.1 *vs.* 4.6, *p* = 0.02) but they required less time for commitment than males (26 *vs.* 42 h/week). There was no significant difference in satisfaction with spouse’s commitment between males and females. Younger married pharmacists with children reported a higher need of family commitment (rho = −0.379, *p* < 0.001). The married pharmacists without children were a bit older than the groups with 1 or more children but it was not significant difference, (*p* = 0.087). Participants who reported having higher than average work challenge had significantly higher need for family commitment (5.1 *vs.* 4.6, *p* = 0.02). Participants who worked part-time reported higher job satisfaction (3.9 *vs.* 3.5, *p* = 0.009). Participants who reported low stress had higher job satisfaction. Interestingly, participants who reported high work challenge reported higher career satisfaction.

High need for family commitment was associated with high work challenge (OR = 6.1, *p* = 0.003), and high workload (OR = 6.1, *p* = 0.010), when controlling for age and gender. Work-related stress, flexibility of work schedule, practice setting, and number of dependents were not significantly associated with high need for family commitment.

**Table 2:** Participants’ demographics and characteristics of married pharmacists.

Variables	Total Number of Responses ^a^	
Age: Mean ± SD	269	48 ± 14
Gender	264	
Female		143 (54%)
Number of dependents: Mean ± SD	240	1.8 ± 1.1
Degree	269	
BS Pharm		166 (62%)
Pharm D		103 (38%)
Full-time pharmacist, *n* (%)	243	182 (75%)
Years of practicing pharmacy since receiving licenses: Mean ± SD	263	23 ± 15
Received advanced training, *n* (%)	269	129 (48%)
Practice settings, *n* (%)	269	
Community—Chain		111 (41%)
Community—Independent		47 (17%)
Hospital		55 (21%)
Others (e.g., nuclear, mail-order, academia, long-term care, *etc.*)		56 (21%)

**^a^** Numbers less than 269 are due to missing data or skipped the family commitment part due to no children.

## 4. Discussion

The pharmacy profession is in a period of significant flux in many aspects. External factors such as substantial healthcare reform, advancing healthcare technologies, and an increasingly aging population have led to an increase in pharmacy services, new professional roles, more job stress and dissatisfaction among pharmacists [9]. According to a recent national survey 45% of U.S. pharmacists believe their current workload has had negative or very negative effects on mental/emotional health [9]. Research suggests that job satisfaction is directly mediated by factors such as work-family conflict, job stress, and career satisfaction and it is influenced by each in varying degrees [7]. In the current study, higher job satisfaction was reported by married pharmacists who work part time. This is consistent with other reports [16,28]. Like others, this study also found that married pharmacists reporting low job stress reported higher job satisfaction [7]. Similarly those who were highly challenged professionally at work reported higher career satisfaction. Collectively, these data and that of others suggest that the practice of pharmacy can be a satisfying career, yet simultaneously stressful on a daily basis [16,29].

**Table 3:** Married participants’ responses on family commitment, job and career satisfaction.

Variables	N ^a^	Need for Family Commitment	N ^a^	Time Needed for Family Commitment (h/week)	N ^a^	Feeling If Spouse Does Not Spend as Much Time Engaged in Family Commitment	N ^a^	Satisfaction with Spouse’s Family Commitment	N ^a^	Job Satisfaction	N ^a^	Career Satisfaction
Gender	196		188		174		190		269		269	
Male	87	4.6 ± 1.6	82	42.0 ± 39.1	80	2.2 ± 0.7	85	4.9 ± 1.3	121	3.6 ± 1.2	121	3.5 ± 1.1
Female	109	5.1 ± 1.3 *	106	26.0 ± 17.3 *	94	2.1 ± 0.6	105	4.7 ± 1.3	143	3.7 ± 1.1	143	3.7 ± 1.1 *
Number of dependents	198		191		176		191		240		240	
0	9	3.3 ± 2.1	8	24.4 ± 18.8	8	1.9 ± 0.6	8	4.8 ± 1.4	39	3.7 ± 1.0	9	3.6 ± 0.9
1	48	5.0 ± 1.2	47	38.1 ± 31.7	44	2.2 ± 0.6	45	5.1 ± 1.0	51	3.7 ± 1.1	1	3.6 ± 1.2
2	88	4.9 ± 1.4	86	31.5 ± 29.3	79	2.1 ± 0.6	87	4.7 ± 1.2	95	3.6 ± 1.2	5	3.6 ± 1.1
3	42	4.9 ± 1.6	39	23.4 ± 16.6	35	2.5 ± 0.6	40	4.6 ± 1.4	44	3.8 ± 1.0	44	3.5 ± 1.1
≥4	11	5.3 ± 0.9	11	57.5 ± 49.0	10	1.8 ± 0.8 *	11	4.5 ± 2.0	11	3.3 ± 1.2	11	3.4 ± 1.0
Degree	199		191		177		193		268		268	
BS Pharm	118	4.7 ± 1.6	111	31.6 ± 31.3	105	2.3 ± 0.6	116	4.7 ± 1.2	165	3.5 ± 1.2	165	3.4 ± 1.1
Pharm D	81	5.2 ± 1.2 *	80	34.2 ± 27.6	72	2.0 ± 0.6 *	77	4.7 ± 1.4	103	3.8 ± 1.1	103	3.9 ± 1.0 *
Type of employment	176		172		156		170		243		243	
Part-time	40	4.9 ± 1.8	36	27.8 ± 28.4	32	2.2 ± 0.7	40	4.9 ± 1.3	61	3.9 ± 1.1	61	3.6 ± 1.1
Full-time	136	4.9 ± 1.4	136	33.6 ± 30.8	124	2.2 ± 0.7	130	4.7 ± 1.2	82	3.5 ± 1.1 *	82	3.6 ± 1.1
Practice settings	199		191		177		193		269		269	
Community—Chain	81	4.8 ± 1.5	78	34.8 ± 28.3	73	2.1 ± 0.7	76	4.7 ± 1.3	111	3.4 ± 1.1	111	3.5 ± 1.1
Community—Independent	32	4.7 ± 1.5	30	32.0 ± 39.6	25	2.3 ± 0.7	33	4.9 ± 1.2	47	3.9 ± 1.1	47	3.6 ± 1.0
Hospital	42	4.8 ± 1.5	40	34.5 ± 33.5	38	2.2 ± 0.6	41	4.7 ± 1.2	55	3.7 ± 1.2	55	3.7 ± 1.0
Others ^b^	44	5.2 ± 1.2	43	27.7 ± 19.4	41	2.3 ± 0.6	43	4.7 ± 1.4	56	3.8 ± 1.1	56	3.5 ± 1.1
Work challenge ^c^	197		189		176		191		266		266	
Low to average	85	4.6 ± 1.5	80	30.4 ± 25.8	73	2.3 ± 0.5	84	4.7 ± 1.3	110	3.5 ± 1.1	110	3.4 ± 1.1
Higher than average	112	5.1 ± 1.4 *	109	34.3 ± 32.6	103	2.1 ± 0.7	107	4.8 ± 1.3	156	3.7 ± 1.1	156	3.7 ± 1.1 *
Stress level ^d^	199		191		181		193		268		268	
Low to average	62	4.8 ± 1.7	56	30.9 ± 30.4	54	2.4 ± 0.7	59	4.8 ± 1.2	86	4.1 ± 1.1	86	3.8 ± 1.1
Higher than average	137	4.9 ± 1.4	135	33.4 ± 29.6	127	2.2 ± 0.7	134	4.7 ± 1.3	82	3.5 ± 1.1 *	182	3.5 ± 1.1

**^a^** Numbers less than 269 are due to missing data; ^b^ Included nuclear pharmacy, mail-order pharmacy, academia, long-term care and others; ^c^ Average of 2.7 (out of 4); ^d^ Average of 2.8 (out of 4); * *p* < 0.05, calculated using Wilcoxon-Mann-Whitney test or Kruskal-Wallis rank test.

Research suggests work is more central to the identity of men, and family is more central to the identity of women [2]. Therefore, it is not surprising the current study suggests that married female pharmacists have a higher need for their spouse to demonstrate a strong personal dedication to be active/involved and participate in the moral and educational development of their children and/or in family activities (*i.e.*, family commitment) than married male pharmacists. According to role theory, roles in each domain result from expectations of others and what is believed to be appropriate behavior for a particular position (e.g., subordinate co-worker, spouse) [2]. Thus, demographic differences such as gender, will result in dissimilar role expectations, role pressures, and subsequent role performance [2]. Our finding may also illustrate an observation of researchers who, in a study of emotion-work among dual-earner couples, found the more frequently that men perceived their partner’s work to be spilling into family life, the less emotion-work they performed relative to their spouse [5]. This study could not determine whether the actual cause of the inequity resulted from men withdrawing support contingent on their spouse’s work-to-family spillover or women increasing their provision of emotion-work to make-up for the conflict caused by the spillover [5]. Our data also demonstrate that married female pharmacists require less time for family commitment than their male counterparts. In the current study the reason why married female pharmacists needed less time while reporting having higher need for family commitment is unclear, but it could be a reflection of their stronger identity in the family domain and a perception among them that stressors in their work domain are mounting.

The need for family commitment among married pharmacists with children was higher than that among married pharmacists without children. This finding could reflect the relationship between role overload and WFC [2]. According to a meta-analytic review the number of children individuals have impacts their ability to accommodate family responsibilities with work demands. Although we did not ascertain the age of the dependents, research suggests younger children typically require more care and resources from their caregivers [2]. Thus, parents with younger children at home report more WFC and have fewer time and energy resources [2]. Our data and these notions may be explained by role theory and resource drain theory [2]. As previously reviewed, according to role theory, roles in each domain result from expectations of others and perceptions of behavior appropriate for a particular position (e.g., subordinate co-worker, spouse) [2]. Resource drain theory posits physical and psychological resources (e.g., time, attention, energy) are finite; thus role stressors that occur in the work and family domains subtract from the finite resources available to the individual [2]. Collectively, both theories imply a positive relationship between role stressors and the work-family conflict [2]. When individuals try to meet the various expectations in each domain, they give in to role pressures. When role pressures are met within role stressors such as role conflict, ambiguity, overload or time demands, resource drain is encountered [2]. Given that an individual has a finite amount of expendable physical and psychological resources, increased role stressors in one domain will increase conflict across both domains. Alternatively, our results may reflect that marital and parental statuses combined may merely be a moderator of each domain and the work-family conflict [2]. That is, married individuals with children are often thought to have more family responsibilities [2]. Thus, when confronted with stressors in the work domain, such individuals should be more likely to recognize them as conflicting with their family domain than married individuals without children [2]. In addition to gender and family factors, age is also recognized as an important determinant of commitment to both family and work domains [1]. Although the difference in age between the married pharmacists with children and the married pharmacists without children was not significant, it is possible that older mean age (50.5 years) observed in the latter group may have contributed in part to the observation that the need for family commitment among married pharmacists with children was higher than that among married pharmacists without children. This finding requires further study.

Those who reported being challenged by their work and having high workload had a significantly higher need for family commitment. These findings are intuitive in that those with more challenge at work and higher workload require a higher need for their spouses to carryout family functions. Thus, pharmacists should discuss the need for family commitment with their spouse. Moreover, employers should be aware that pharmacists in high challenge and high workload positions may have increased need for family commitment.

This study has limitations. First, the participants were drawn from only south central region of the U.S. Their perspectives on family commitment, work characteristics and satisfaction may not be generalized to pharmacists in other regions or countries. Many respondents were also in the middle age group. The findings may not be similar to younger or older generations of pharmacists. Secondly, the study was a cross-sectional design, which is not be able to identify cause and effect relationships. Thus, we reported the findings as associations among family commitment, work characteristics, and satisfaction. Another limitation was we measured workload, work challenge, and work-related stress based on only one item. The associations should be verified in future research. Although there were no statistically significant differences in demographics between early and late respondents in the full sample [23], the subsample used for this study had less female than male in the late response group, compared to the early group (44% *vs.* 59%, *p* = 0.02). The scores for the need for family commitment were also significantly lower in the late response group, compared to early response group (4.5 *vs.* 5, *p* = 0.03). We believe that the lower scores for the need for family commitment were due to having less females in the late response group, compared to the early response group. Non-response bias could potentially be present in findings. Finally, our sample size was relatively small but we reached to 97% of needed sample size. Although a stratified random sampling provided a confidence that the sample represented pharmacists in the study region, the future research with a bigger sample size is warranted to confirm the findings from our research.

## 5. Conclusions

Organizations have a clear role in ensuring a healthy work-life balance for employees. Being a pharmacist is a high stress and high challenge profession. Our study found that higher work challenge and workload were associated with higher need for family commitment among married pharmacists. Stress and work challenge are associated with pharmacist satisfaction in their job and career. To help keep their pharmacists in the workforce, employers should recognize how the work environment contributes to the work-family conflict and develop strategies to help their pharmacists balance their work and family domains. In addition, pharmacists could seek the aid of support groups to help alleviate stress and provide the support they need. Lastly, the impact of work–family interactions on pharmacy career satisfaction should be further investigated.
